# The role of bilirubin as a biomarker of rheumatic diseases: a systematic review and meta-analysis

**DOI:** 10.3389/fimmu.2024.1369284

**Published:** 2024-06-14

**Authors:** Angelo Zinellu, Arduino A. Mangoni

**Affiliations:** ^1^ Department of Biomedical Sciences, University of Sassari, Sassari, Italy; ^2^ Discipline of Clinical Pharmacology, College of Medicine and Public Health, Flinders University, Adelaide, SA, Australia; ^3^ Department of Clinical Pharmacology, Flinders Medical Centre, Southern Adelaide Local Health Network, Adelaide, SA, Australia

**Keywords:** bilirubin, oxidative stress, autoinflammatory diseases, mixed-pattern diseases, autoimmune diseases, rheumatic diseases, biomarkers, inflammation

## Abstract

The identification of novel, yet easily measurable biomarkers of inflammation and oxidative stress might assist in the diagnosis and management of patients with rheumatic diseases (RDs). We conducted a systematic review and meta-analysis of studies investigating the circulating concentrations of bilirubin, the end product of heme metabolism and a potent endogenous antioxidant with anti-inflammatory properties, in patients with RDs and healthy controls. The electronic databases PubMed, Scopus, and Web of Science were searched from inception to 31 December 2023 for relevant articles. We evaluated the risk of bias and the certainty of evidence using the Joanna Briggs Checklist and the Grades of Recommendation, Assessment, Development, and Evaluation Working Group system, respectively. In 17 eligible studies, all with low risk of bias, compared to controls, patients with RDs had significantly lower concentrations of total bilirubin (standard mean difference, SMD=-0.68, 95% CI -0.91 to -0.44, p<0.001; I^2^ = 92.5%, p<0.001; low certainty of evidence), direct (conjugated) bilirubin (SMD=-0.67, 95% CI -0.92 to -0.41, p<0.001; I^2^ = 81.7%, p<0.001; very low certainty of evidence), and the active antioxidant and anti-inflammatory indirect (unconjugated) form of bilirubin (SMD=-0.71, 95% CI -1.18 to -0.24, p=0.003; I^2^ = 95.1%, p<0.001; very low certainty of evidence). The results of the meta-analysis were stable in sensitivity analysis. In meta-regression, there were no significant associations between the SMD of total bilirubin and several clinical and demographic characteristics, including age, male to female ratio, number of participants, liver enzymes and erythrocyte sedimentation rate. In subgroup analysis, the SMD of total bilirubin was significant across a range of RDs, including rheumatoid arthritis, systemic lupus erythematosus, primary Sjögren syndrome, and myositis. Therefore, the results of our systematic review and meta-analysis suggests that the reductions in bilirubin concentrations observed in patients with RDs reflect a state of impaired antioxidant and anti-inflammatory defence due to bilirubin consumption and highlight the promising role of this endogenous product as a biomarker of RDs.

**Systematic review registration:**

https://www.crd.york.ac.uk/prospero/, identifier CRD42023500649.

## Introduction

The term “rheumatic diseases (RDs)” includes a range of chronic, disabling conditions that are characterized by a pro-inflammatory and pro-oxidant state involving the musculoskeletal system and other organ and tissues. RDs are predominantly autoimmune (e.g., progressive systemic sclerosis, rheumatoid arthritis, systemic lupus erythematosus, and Sjogren’s syndrome), mixed-autoimmune-autoinflammatory (e.g., ankylosing spondylitis, axial spondylarthritis, psoriatic arthritis, and Behcet’s disease), or autoinflammatory (e.g., familial Mediterranean fever) (1, 2).

The robust evidence of a dysregulation of inflammatory pathways and redox balance mechanisms in RDs has led to the routine use of circulating biomarkers of inflammation, e.g., C-reactive protein (CRP), erythrocyte sedimentation rate (ESR), and ferritin, to diagnose the presence of specific RDs (3–6). However, their limited diagnostic accuracy in several types of RDs has stimulated a significant body of research to identify better biomarkers (3, 7–9). Furthermore, the analytical challenges associated with the routine measurement of biomarkers of oxidative stress (10–12) has stimulated further research to identify the potential utility of other, more stable, and easily measurable endogenous compounds. One such compound is bilirubin, the product of heme metabolism which has been shown to exert potent endogenous antioxidant with anti-inflammatory effects in experimental and human studies (13–16). Physiologically, approximately 80% of bilirubin is generated from the breakdown of hemoglobin in senescent red blood cells and from erythroid cells undergoing premature destruction in the bone marrow (17). The rest is generated from the breakdown of several heme-containing proteins primarily located in the liver and muscle (17). In the blood, bilirubin is bound to albumin (unconjugated or indirect bilirubin) or conjugated with glucuronic acid (conjugated or direct bilirubin) and can be measured in either form as well as total bilirubin (17, 18). It is important however to emphasize that the reported anti-inflammatory and antioxidant effects of bilirubin are primarily attributed to the unconjugated or indirect form of bilirubin (19–22).

Given the increasing interest in the potential clinical and diagnostic utility of bilirubin in RDs, we conducted a systematic review and meta-analysis of studies investigating this endogenous antioxidant with anti-inflammatory properties in patients with RDs and healthy controls. We speculated that the presence of RDs was associated with a significant reduction in bilirubin concentrations, particularly the unconjugated or indirect form vs. healthy controls. We also investigated the presence of possible associations between the effect size of the between-group differences in bilirubin concentrations and several relevant demographic and clinical parameters, including specific types of RDs, age, male to female ratio, conventional biomarkers of inflammation (erythrocyte sedimentation rate, ESR), standard liver function enzymes (alanine aminotransferase, ALT, and aspartate aminotransferase, AST), study location, and study design.

## Materials and methods

### Search strategy and study selection

We conducted a systematic search of publications in the electronic databases PubMed, Web of Science, and Scopus from inception to 31 December 2023 using the following terms and their combination: “bilirubin” AND “rheumatic diseases” OR “rheumatoid arthritis” OR “psoriatic arthritis” OR “ reactive arthritis” OR “ankylosing spondylitis” OR “systemic lupus erythematosus” OR “systemic sclerosis” OR “scleroderma” OR “Sjogren’s syndrome” OR “connective tissue diseases” OR “vasculitis” OR “Behçet’s disease” OR “idiopathic inflammatory myositis” OR “polymyositis” OR “dermatomyositis” OR “gout” OR “pseudogout” OR “systemic vasculitis” OR “ANCA-associated vasculitis” OR “Takayasu arteritis” OR “polyarteritis nodosa” OR “osteoarthritis” OR “fibromyalgia” OR “granulomatous polyangiitis” OR “Henoch-Schonlein purpura” OR “Wegener granulomatosis”. Each abstract was independently screened by two investigators. If relevant, the two investigators independently reviewed the full articles according to the following inclusion criteria: (i) the assessment of bilirubin concentrations in patients with RDs and healthy controls (case-control design), (ii) the inclusion of participants aged ≥18 years, (iii) the use of English language, and (iv) the availability of full-text. The references of each article were also hand searched for additional studies.

The following qualitative and quantitative variables were independently extracted and transferred to an electronic spreadsheet for analysis: year of publication, first author, study design (prospective or retrospective), study country, type of RD, sample size, age, male to female ratio, total bilirubin, direct bilirubin, indirect bilirubin, alanine aminotransferase (ALT), aspartate aminotransferase (AST), and erythrocyte sedimentation rate (ESR).

The risk of bias was assessed using the Joanna Briggs Institute (JBI) Critical Appraisal Checklist for analytical studies (23). The risk was considered low, moderate, and high for studies addressing ≥75%, ≥50% and <75%, and <50% of checklist items, respectively. The certainty of evidence was assessed using the Grades of Recommendation, Assessment, Development and Evaluation (GRADE) Working Group system (24). The study complied with the Preferred Reporting Items for Systematic reviews and Meta-Analyses (PRISMA) 2020 statement (**Supplementary Tables 1**, **2**) (25). The study protocol was registered in the International Prospective Register of Systematic Reviews (PROSPERO registration number: CRD42023500649).

### Statistical analysis

Standardized mean differences (SMDs) and 95% confidence intervals (CIs) were used to generate forest plots of continuous data and to assess differences in bilirubin concentrations between patients with RDs and healthy controls. A p-value <0.05 indicated statistical significance. If required, the mean and standard deviation values were extrapolated from medians and interquartile ranges or medians and ranges using published methods (26). The heterogeneity of the SMD across studies was tested by using the Q statistic (significance level at p<0.10) and was interpreted as low when I^2^ ≤25%, moderate when 25%< I^2^ <75%, and high when I^2^ ≥75% (27, 28). A random-effect model based on the inverse-variance method was used in the presence of high heterogeneity. Sensitivity analysis was conducted to confirm the stability of the results (29). The presence of publication bias was assessed using the Begg’s and the Egger’s tests (30, 31), with a p-value <0.05 set as the level of significance, and the Duval and Tweedie “trim-and-fill” method (32).

Univariate meta-regression analyses were conducted to investigate associations between the effect size and the following parameters: year of publication, study design, country where the study was conducted, type of RD, sample size, age, male to female ratio, ALT, AST and ESR. Statistical analyses were performed using Stata 14 (Stata Corp., College Station, TX, USA).

## Results

### Study selection

**Figure 1:**
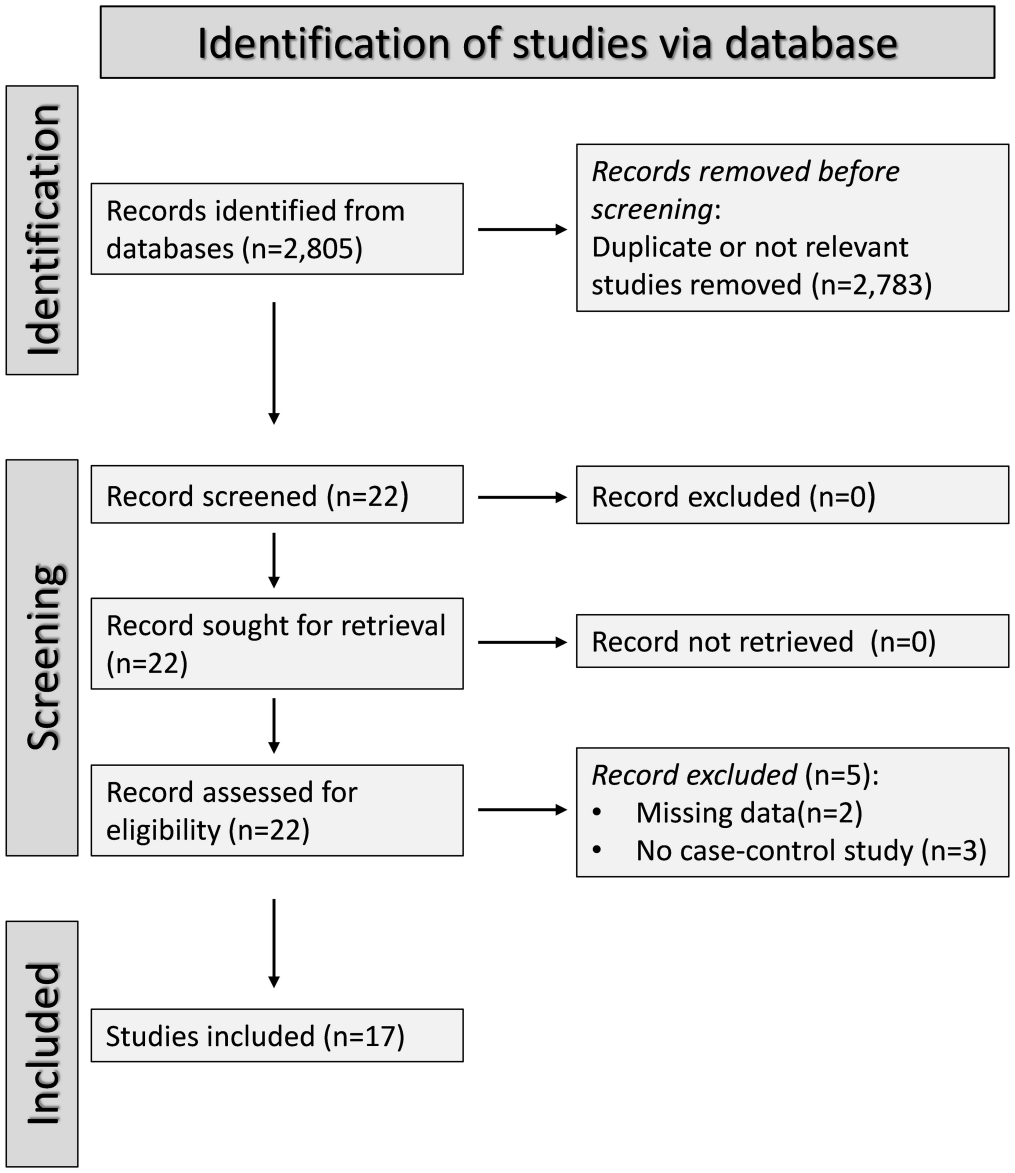
PRISMA 2020 flow diagram.

A flow chart describing the screening process is described in **Figure 1**. From a total of 2,805 articles initially identified, 2,783 were excluded because they were either duplicates or irrelevant. After a full-text review of the remaining 22 articles, a further two were excluded because of missing data and three because they did not have a case-control design, leaving 17 studies for further analysis (33–49) (**Figure 1**; **Table 1**). The risk of bias was considered low in all studies (**Table 2**). The initial certainty of evidence was also considered low because of the cross-sectional design of the studies selected.

**Table 1:** Characteristics of the studies investigating bilirubin in patients with rheumatic diseases and healthy controls.

Study	Healthy controls				Patients with rheumatic diseases				Disease type	Study design
	n	Age (Years)	M/F	Total bilirubin Direct bilirubin Indirect bilirubin (Mean ± SD)	n	Age (Years)	M/F	Total bilirubin Direct bilirubin Indirect bilirubin (Mean ± SD)		
Vitek L et al., 2010, Czech Republic (33)	180	39	93/87	12.53 ± 5.6 NR NR	218	39	32/186	7.03 ± 2.54 NR NR	SLE	P
Yang Z et al., 2012, China (34)	154	39	17/137	13.42 ± 5.43 2.75 ± 0.94 10.67 ± 4.49	179	36	22/157	8.25 ± 3.92 2.00 ± 1.49 6.00 ± 2.99	SLE	P
Peng YF et al., 2016, China (35)	108	45	34/74	15.2 ± 2.97 NR NR	77	47	22/55	11.7 ± 4.37 NR NR	PM	R
Smirnova SV et al., 2016, Russia (36)	30	NR	NR	12.13 ± 2.41 NR NR	98	NR	NR	15.77 ± 4.82 NR NR	PsA	P
van Bon L et al., 2016, USA (37)	20	NR	NR	0.62 ± 0.19 NR NR	41	NR	NR	0.47 ± 0.14 NR NR	SSc	P
Chen Z et al., 2017, China (38)	274	50	80/194	13.45 ± 4.7 3.81 ± 1.50 9.63 ± 3.65	110	51	34/76	8.31 ± 2.93 3.29 ± 1.22 5.03 ± 2.06	PM/DM	NR
Junping D et al. (a) 2017, China (39)	96	40	52/44	11.4 ± 3.01 4.17 ± 0.98 7.23 ± 2.26	35	59	8/27	7.57 ± 2.16 2.47 ± 1.08 5.13 ± 2.09	RA	R
Junping D et al. (b) 2017, China (39)	96	40	52/44	11.4 ± 3.01 4.17 ± 0.98 7.23 ± 2.26	81	56	47/34	10.73 ± 4.14 3.40 ± 1.43 7.50 ± 3.09	OA	R
Peng YF et al., 2017, China (40)	193	29	10/183	0.7 ± 0.22 NR NR	115	28	5/110	0.6 ± 0.31 NR NR	TA	P
Peng YF et al., 2017, China (41)	346	50	60/286	10.3 ± 4.16 NR NR	173	52	37/136	8.9 ± 3.99 NR NR	RA	P
Koca TT et al., 2018, Turkey (42)	75	46	44/31	0.53 ± 0.17 0.19 ± 0.61 0.33 ± 0.17	71	42	40/31	0.5 ± 0.25 0.0016 ± 0.008 0.39 ± 0.20	BD	R
Koca TT et al., 2019, Turkey (43)	75	52	40/35	0.52 ± 0.22 0.19 ± 0.01 0.37 ± 0.17	75	50	32/43	0.53 ± 0.22 0.15 ± 0.07 0.33 ± 0.17	RA	NR
Xie J et al., 2020, China (44)	100	51	17/83	13.67 ± 6.02 NR NR	97	54	12/12	9.67 ± 4.51 NR NR	pSS	R
Zhang Z et al., 2020, China (45)	138	48	17/121	10.88 ± 3.09 2.84 ± 1.15 7.93 ± 2.92	116	47	13/103	6.89 ± 3.3 2.32 ± 1.34 4.53 ± 2.53	pSS	R
Wang J et al., 2021, China (46)	78	40	55/23	16.15 ± 4.86 NR NR	78	41	51/27	9.97 ± 5.95 NR NR	axial SpA	P
Zhang W et al., 2023, China (47)	332	49	0/332	13.43 ± 4.62 NR NR	341	40	0/341	11 ± 4.1 NR NR	SLE	P
Wang X et al., 2023, China (48)	73	44	38/35	10.1 ± 2.8 NR 10.1 ± 2.8	68	44	44/24	6.9 ± 3.7 NR 6.9 ± 3.7	PsA	R
Zhang H et al., 2023, China (49)	197	50	35/162	13.77 ± 3.66 NR NR	197	50	35/162	11.22 ± 3.92 NR NR	RA	P

BD, Behcet disease; DM, dermatomyositis; M/F, male to female ratio; NR, not reported; OA, osteoarthritis; P, prospective; PM, polymyositis; PsA, psoriatic arthritis; pSS, primary Sjögren syndrome; R, retrospective; RA, rheumatoid arthritis; SLE, systemic lupus erythematosus; SpA, spondyloarthritis; SSc, systemic sclerosis; TA, Takayasu arteritis. Bilirubin values are reported as µmol/L or mg/dL.

**Table 2:** Assessment of the risk of bias using the Joanna Briggs Institute critical appraisal checklist.

Study	Were the inclusion criteria clearly defined?	Were the subjects and the setting described in detail?	Was the exposure measured in a reliable way?	Were standard criteria used to assess the condition?	Were confounding factors identified?	Were strategies to deal with confounding factors stated?	Were the outcomes measured in a reliable way?	Was appropriate statistical analysis used?	Risk of bias
Vitek L et al. (33)	Yes	Yes	Yes	Yes	Yes	Yes	Yes	Yes	Low
Yang Z et al. (34)	Yes	Yes	Yes	Yes	Yes	Yes	Yes	Yes	Low
Peng YF et al. (35)	Yes	Yes	Yes	Yes	Yes	Yes	Yes	Yes	Low
Smirnova SV et al. (36)	Yes	Yes	Yes	Yes	No	No	Yes	Yes	Low
van Bon L et al. (37)	Yes	Yes	Yes	Yes	No	No	Yes	Yes	Low
Chen Z et al. (38)	Yes	Yes	Yes	Yes	Yes	Yes	Yes	Yes	Low
Junping D et al. (39)	Yes	Yes	Yes	Yes	Yes	Yes	Yes	Yes	Low
Peng YF et al. (41)	Yes	Yes	Yes	Yes	Yes	Yes	Yes	Yes	Low
Peng YF et al. (40)	Yes	Yes	Yes	Yes	Yes	Yes	Yes	Yes	Low
Koca TT et al. (42)	Yes	Yes	Yes	Yes	No	No	Yes	Yes	Low
Koca TT et al. (43)	Yes	Yes	Yes	Yes	No	No	Yes	Yes	Low
Xie J et al. (44)	Yes	Yes	Yes	Yes	Yes	Yes	Yes	Yes	Low
Zhang Z et al. (45)	Yes	Yes	Yes	Yes	Yes	Yes	Yes	Yes	Low
Wang J et al. (46)	Yes	Yes	Yes	Yes	No	No	Yes	Yes	Low
Zhang W et al. (47)	Yes	Yes	Yes	Yes	Yes	Yes	Yes	Yes	Low
Wang X et al. (48)	Yes	Yes	Yes	Yes	Yes	Yes	Yes	Yes	Low
Zhang H et al. (49)	Yes	Yes	Yes	Yes	Yes	Yes	Yes	Yes	Low

### Total bilirubin and rheumatic diseases

Sixteen studies including 17 group comparators investigated total bilirubin in a total of 2,102 patients with RDs (mean age 44 years, 80% females) and 2,492 healthy controls (mean age 45 years, 75% females) (33–47, 49). Eleven studies were conducted in China (34, 35, 38–41, 44–47, 49), two in Turkey (42, 43), one in the Czech Republic (33), one in Russia (36), and one in USA (37). Four study groups included patients with rheumatoid arthritis (39, 41, 43, 49), three with systemic lupus erythematosus (33, 34, 47), two with primary Sjögren syndrome (44, 45), one with psoriatic arthritis (36), one with systemic sclerosis (37), one with osteoarthritis (39), one with Takayasu arteritis (40), one with Behcet disease (42), one with axial spondyloarthritis (46), one with polymyositis (35), and one with both polymyositis and dermatomyositis (38). The study design was prospective in nine studies (33, 34, 36, 37, 40, 41, 46, 47, 49), retrospective in five (35, 39, 42, 44, 45), and unknown in the remaining two (38, 43).

**Figure 2:**
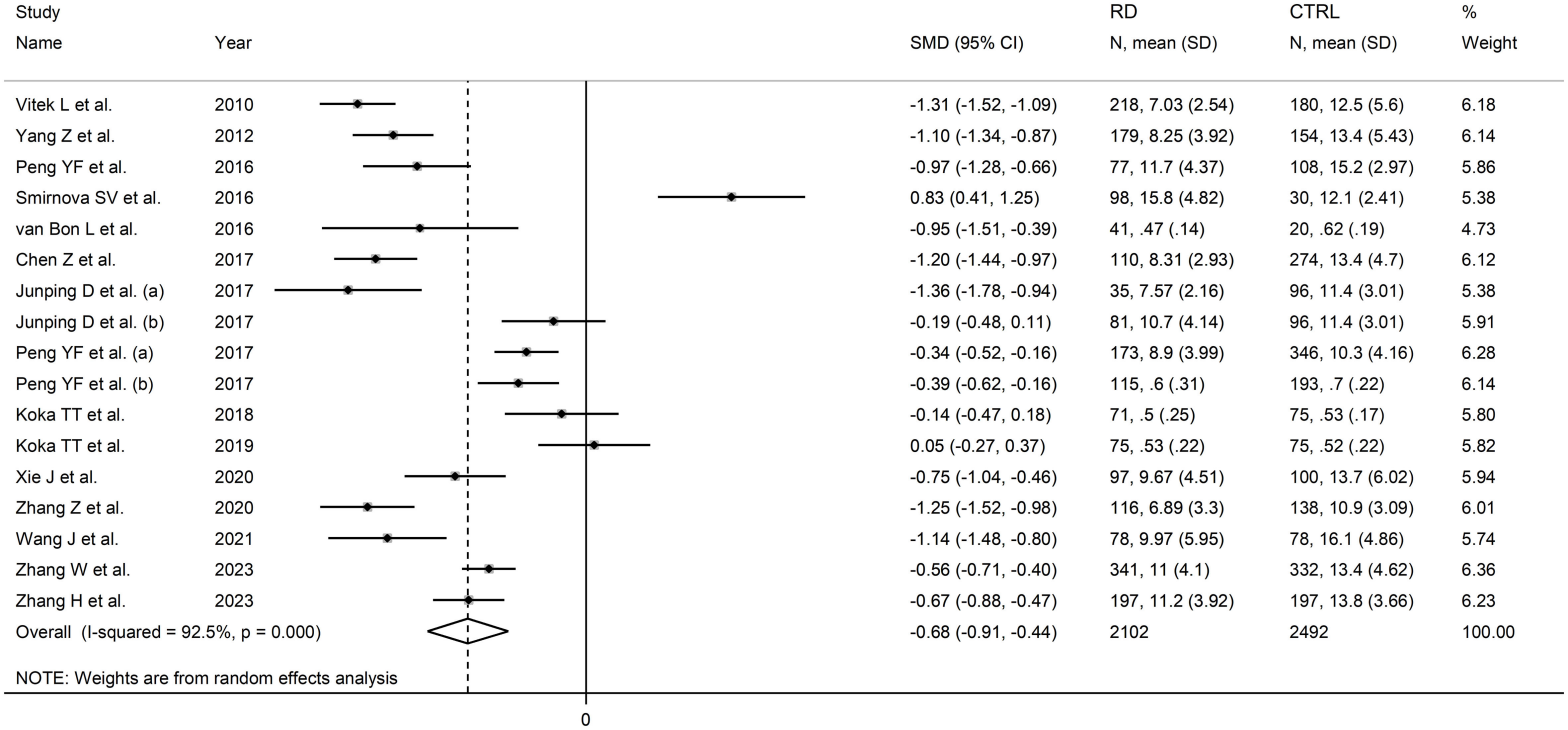
Forest plot of studies investigating total bilirubin in healthy controls and patients with rheumatic diseases.

**Figure 3:**
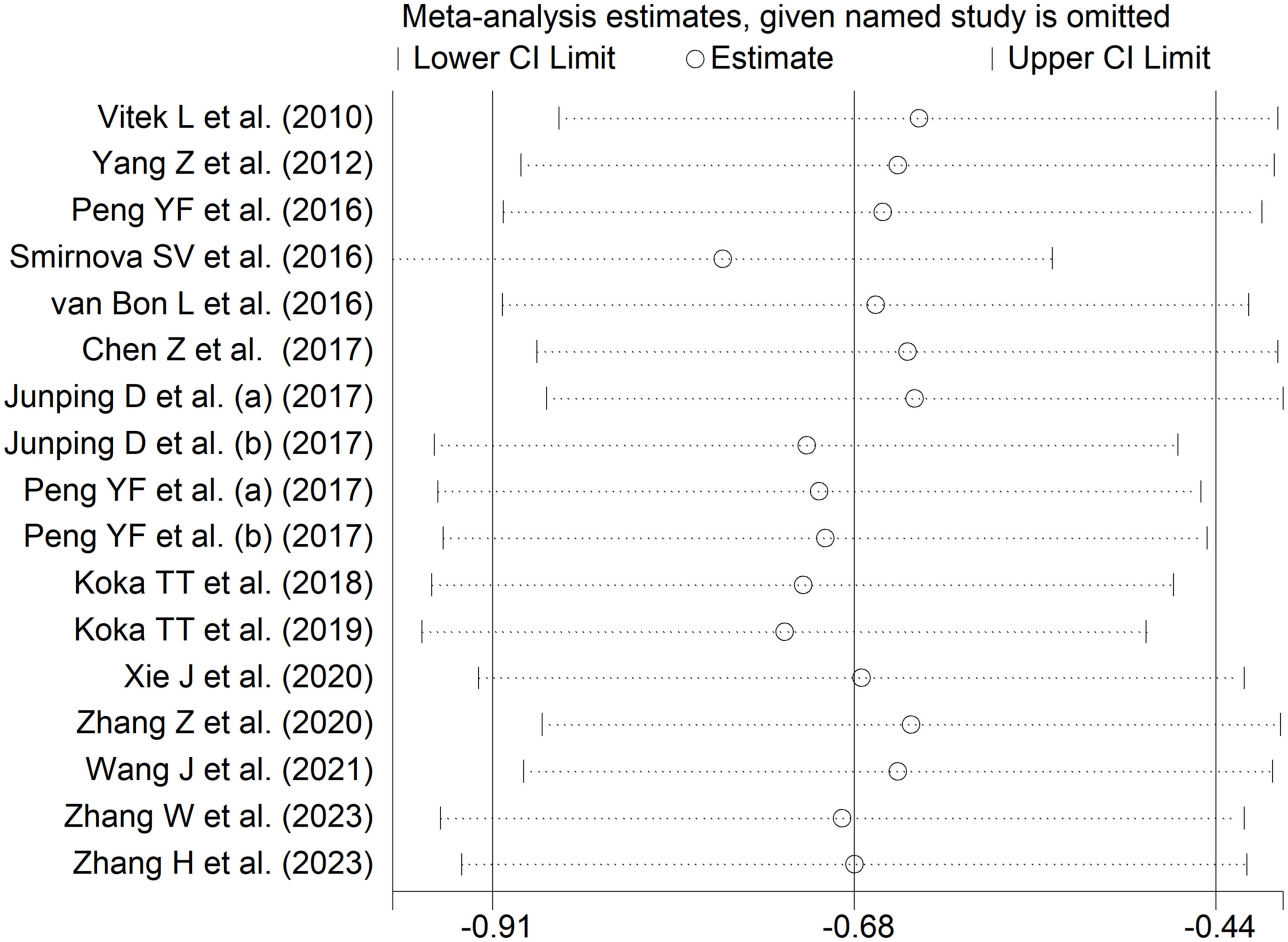
Sensitivity analysis of the association between total bilirubin and rheumatic diseases.

**Figure 4:**
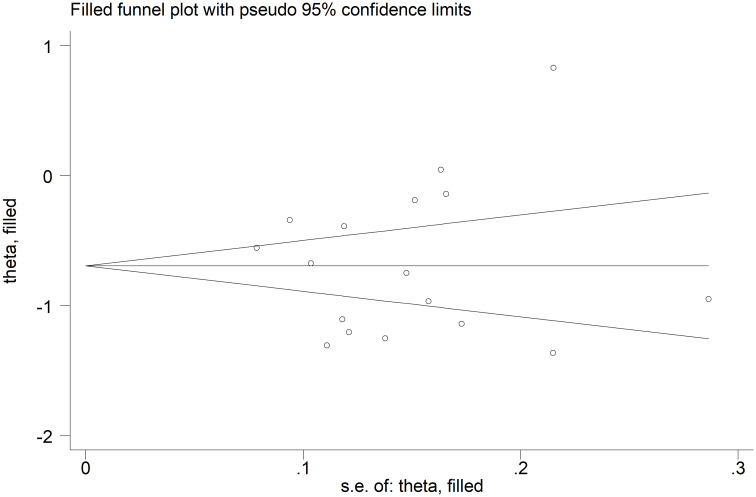
Funnel plot of studies investigating the association between total bilirubin and rheumatic diseases after “trimming-and-filling”. Dummy studies and genuine studies are represented by enclosed circles and free circles, respectively.

The forest plot showed that the total bilirubin concentrations in patients with RDs were significantly lower when compared to controls (SMD=-0.68, 95% CI -0.91 to -0.44, p<0.001; I^2^ = 92.5%, p<0.001; **Figure 2**). Sensitivity analysis showed stability of the results, with the corresponding pooled SMD values ranging between -0.76 and -0.64 (**Figure 3**). There was no evidence of publication bias according to the Begg’s (p=0.59) or the Egger’s (p=0.88) test. The “trim-and-fill” method did not identify any missing study to be added to the funnel plot to ensure symmetry (**Figure 4**).

**Figure 5:**
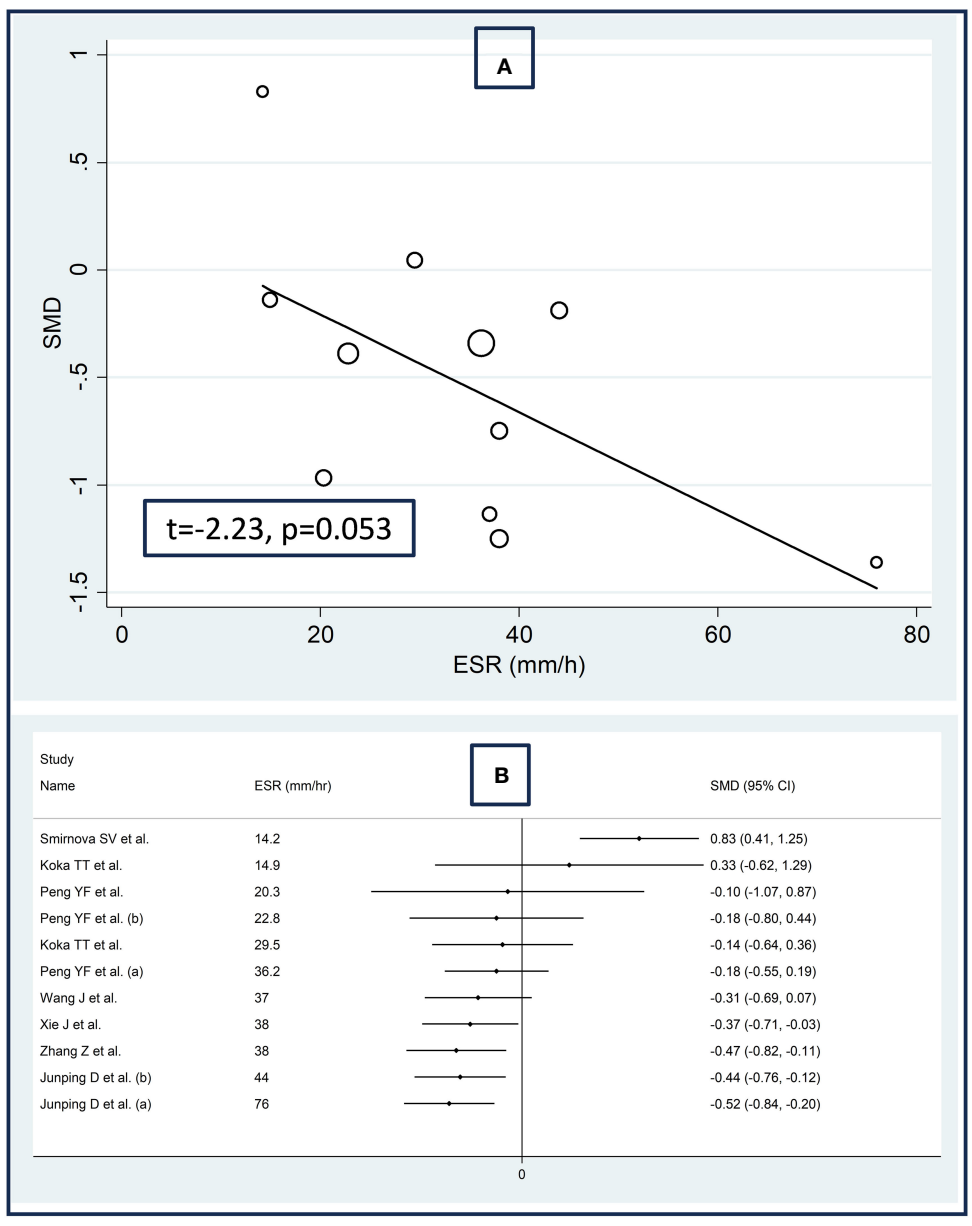
Bubble plot reporting the univariate meta-regression analysis between the effect size and erythrocyte sedimentation rate **(A)** and cumulative meta-analysis of total bilirubin concentrations based on the erythrocyte sedimentation rate **(B)**.

There were no significant associations between the effect size and age (t=0.56, p=0.59), male to female ratio (t=1.51, p=0.16), sample size (t=-0.49, p=0.63), AST (t=0.19, p=0.85), or ALT (t=-0.39, p=0.71) in univariate meta-regression analysis. A non-significant trend was observed between the effect size and ESR (t=-2.23, p=0.053, **Figure 5A**). This relationship was even more evident by cumulative analysis based on the ESR values of patients with RDs performed by metacum command (**Figure 5B**).

**Figure 6:**
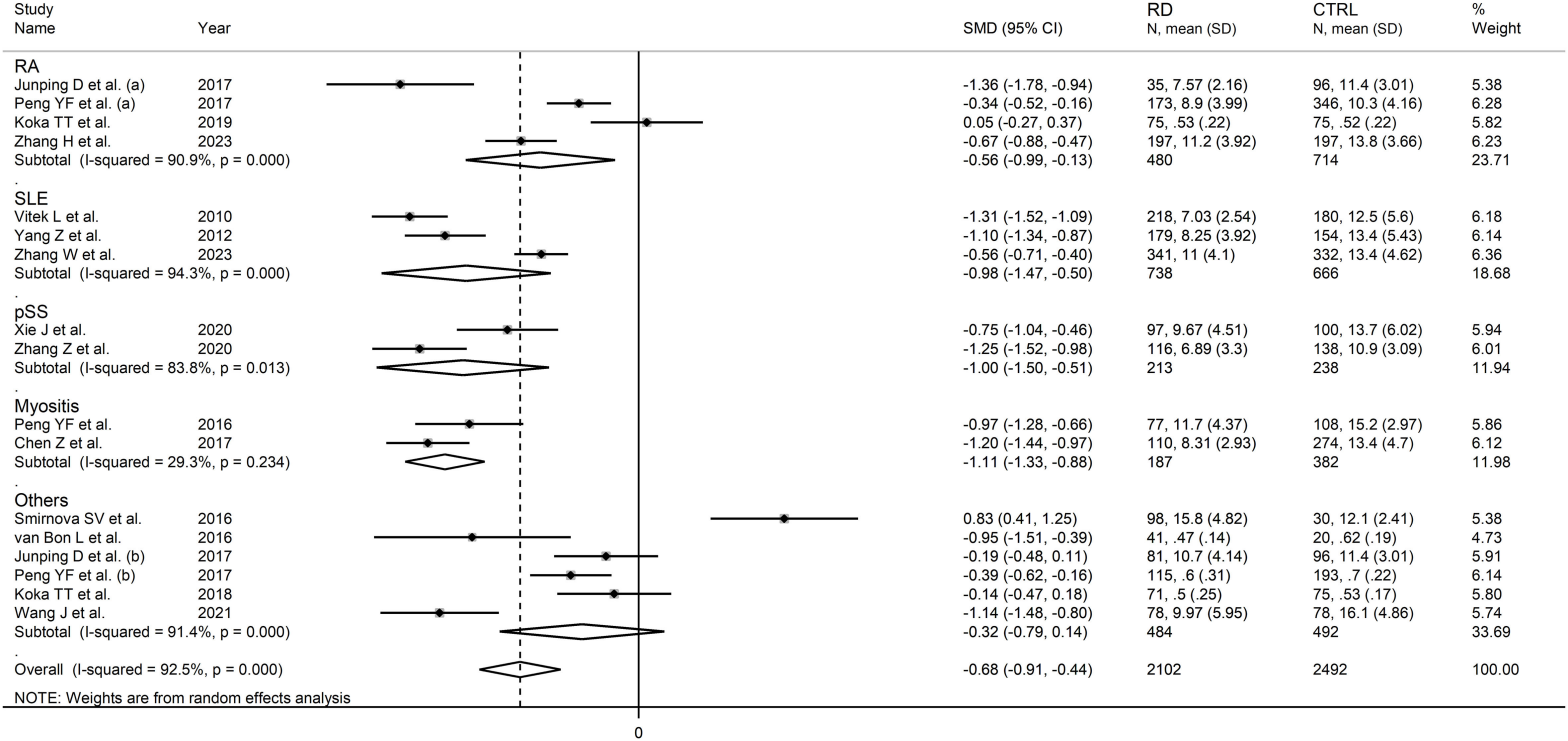
Forest plot of studies investigating total bilirubin according to the type of rheumatic disease.

**Figure 7:**
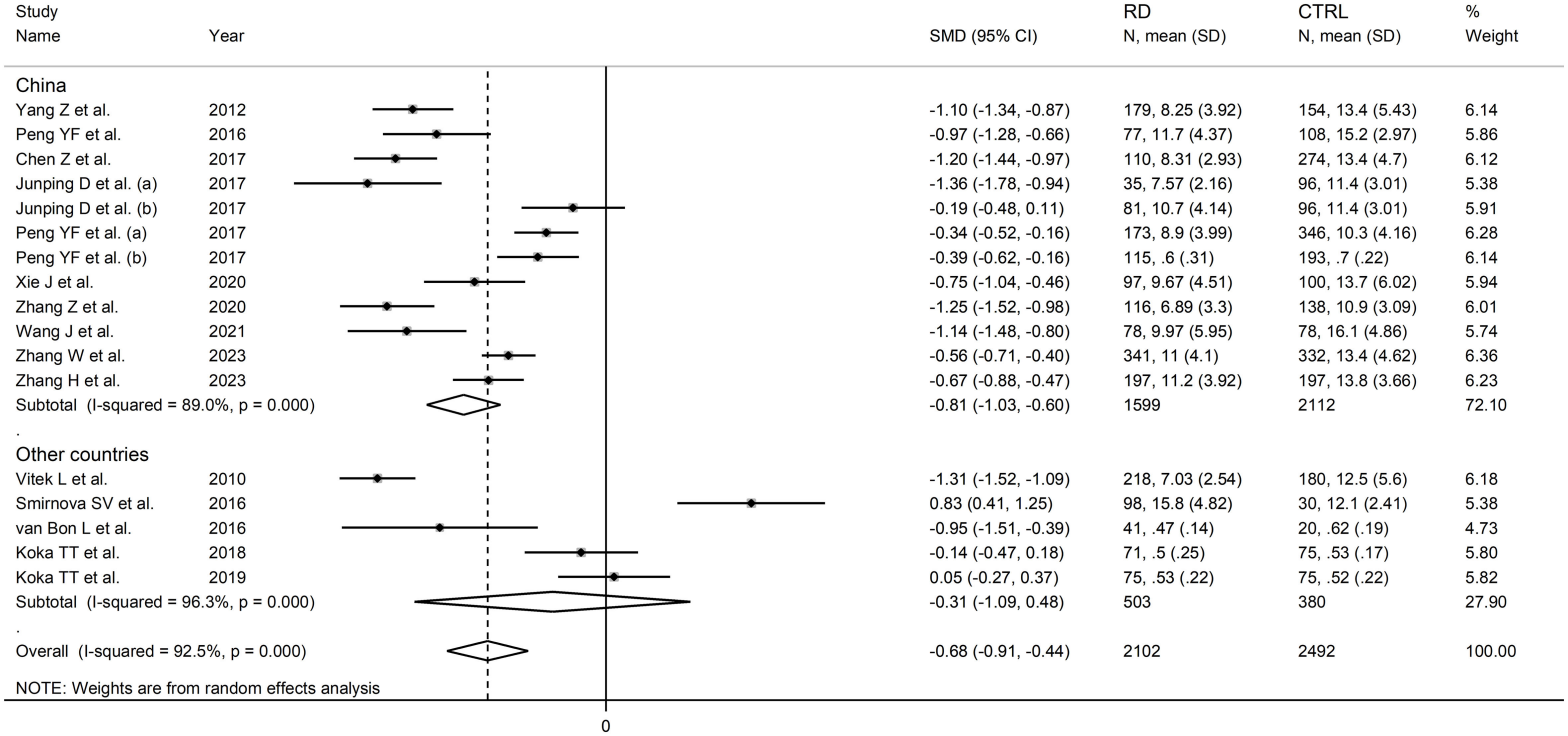
Forest plot of studies investigating total bilirubin according to the study country.

**Figure 8:**
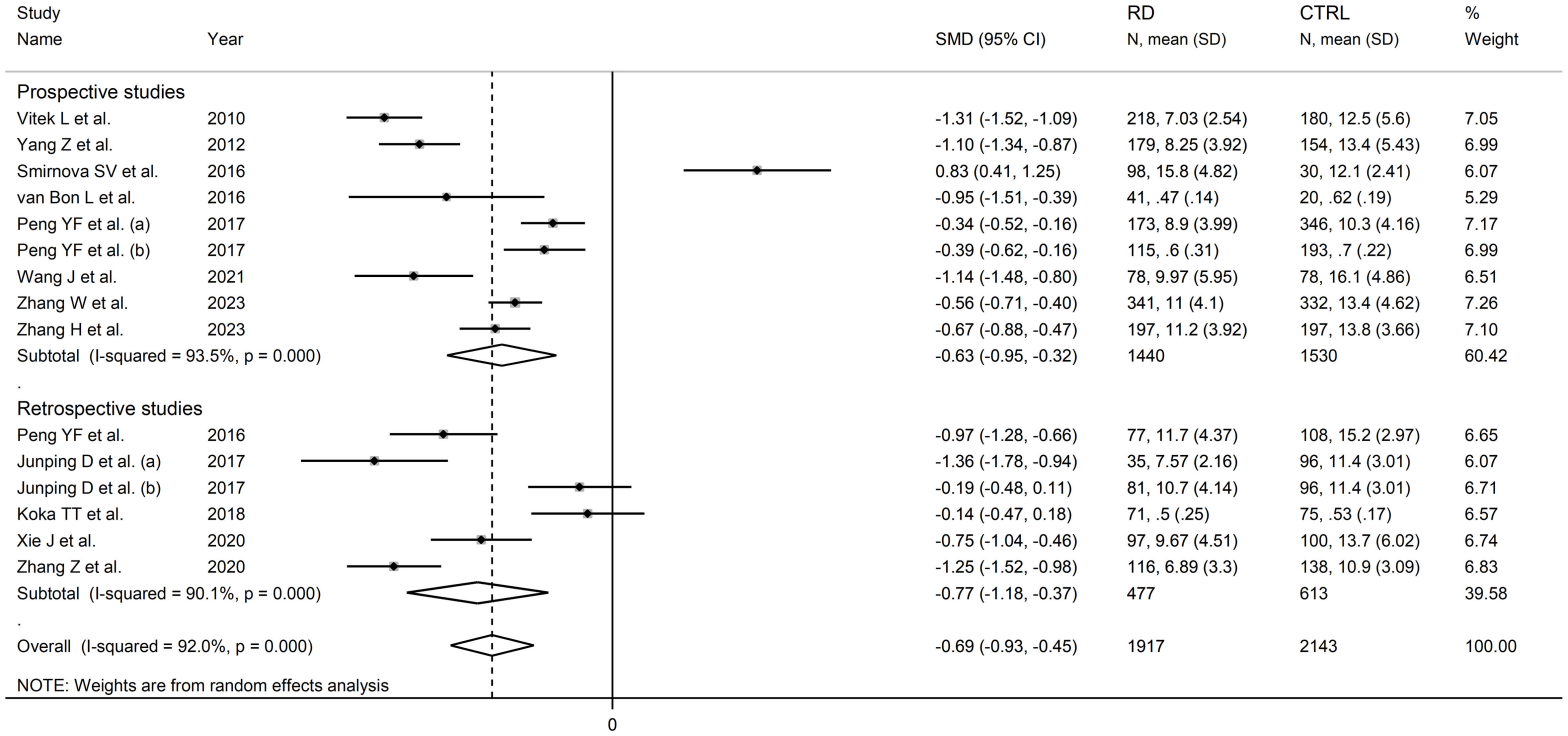
Forest plot of studies investigating total bilirubin according to study design.

In subgroup analysis, the pooled SMD was significant in studies in patients with rheumatoid arthritis (SMD=-0.56, 95% CI -0.99 to -0.13, p=0.01; I^2^ = 90.9%, p<0.001), systemic lupus erythematosus (SMD=-0.98, 95% CI -1.47 to -0.50, p<0.001; I^2^ = 94.3%, p<0.001), primary Sjögren syndrome (SMD=-1.00, 95% CI -1.50 to -0.51, p<0.001; I^2^ = 83.8%, p<0.001) and myositis (SMD=-1.11, 95% CI -1.33 to -0.88 p<0.001; I^2^ = 29.3%, p=0.234, **Figure 6**), with a relatively lower between study variance in the myositis subgroup. The pooled SMD was statistically significant in studies conducted in China (SMD=-0.81, 95% CI -1.03 to -0.60, p<0.001; I^2^ = 89.0%, p<0.001) but not in other countries (SMD=-0.31, 95% CI -1.09 to 0.48, p=0.45; I^2^ = 96.3%, p<0.001; **Figure 7**). Furthermore, the pooled SMD was statistically significant both in prospective (SMD=-0.63, 95% CI -0.95 to -0.32, p<0.001; I^2^ = 93.5%, p<0.001) and retrospective studies (SMD=-0.77, 95% CI -1.18 to -0.37, p<0.001; I^2^ = 90.1%, p<0.001; **Figure 8**).

The overall level of certainty remained low (rating 2) after considering the low risk of bias in all studies (no change), the high but partly explainable heterogeneity (no change), the lack of indirectness (no change), the moderate effect size (SMD=-0.68) (50), and the absence of publication bias (no change).

### Direct (conjugated) bilirubin and rheumatic diseases

Six studies including seven comparator groups investigated direct bilirubin concentrations in a total of 667 patients with RDs (mean age 46 years, 70% females) and 908 healthy controls (mean age 46 years, 67% females) (34, 38, 39, 42, 43, 45). Four studies were conducted in China (34, 38, 39, 45), and two in Turkey (42, 43). Two study groups included individuals with rheumatoid arthritis (39, 43), one with systemic lupus erythematosus (34), one with myositis (38), one with osteoarthritis (39), one with Behcet disease (42), and one with primary Sjögren syndrome (45). The study design was retrospective in three studies (39, 42, 45), prospective in one (34), and unknown in the remaining two (38, 43).

**Figure 9:**
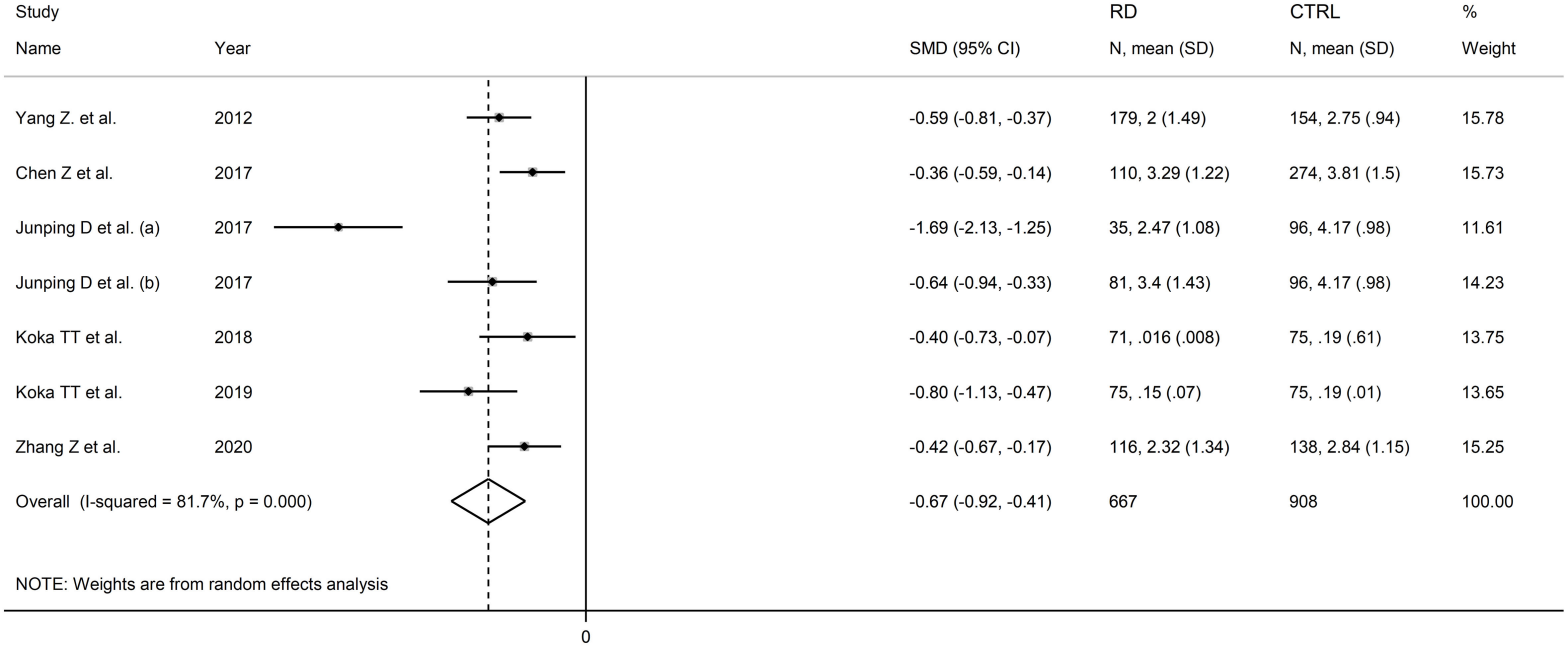
Forest plot of studies investigating direct bilirubin in healthy controls and patients with rheumatic diseases.

**Figure 10:**
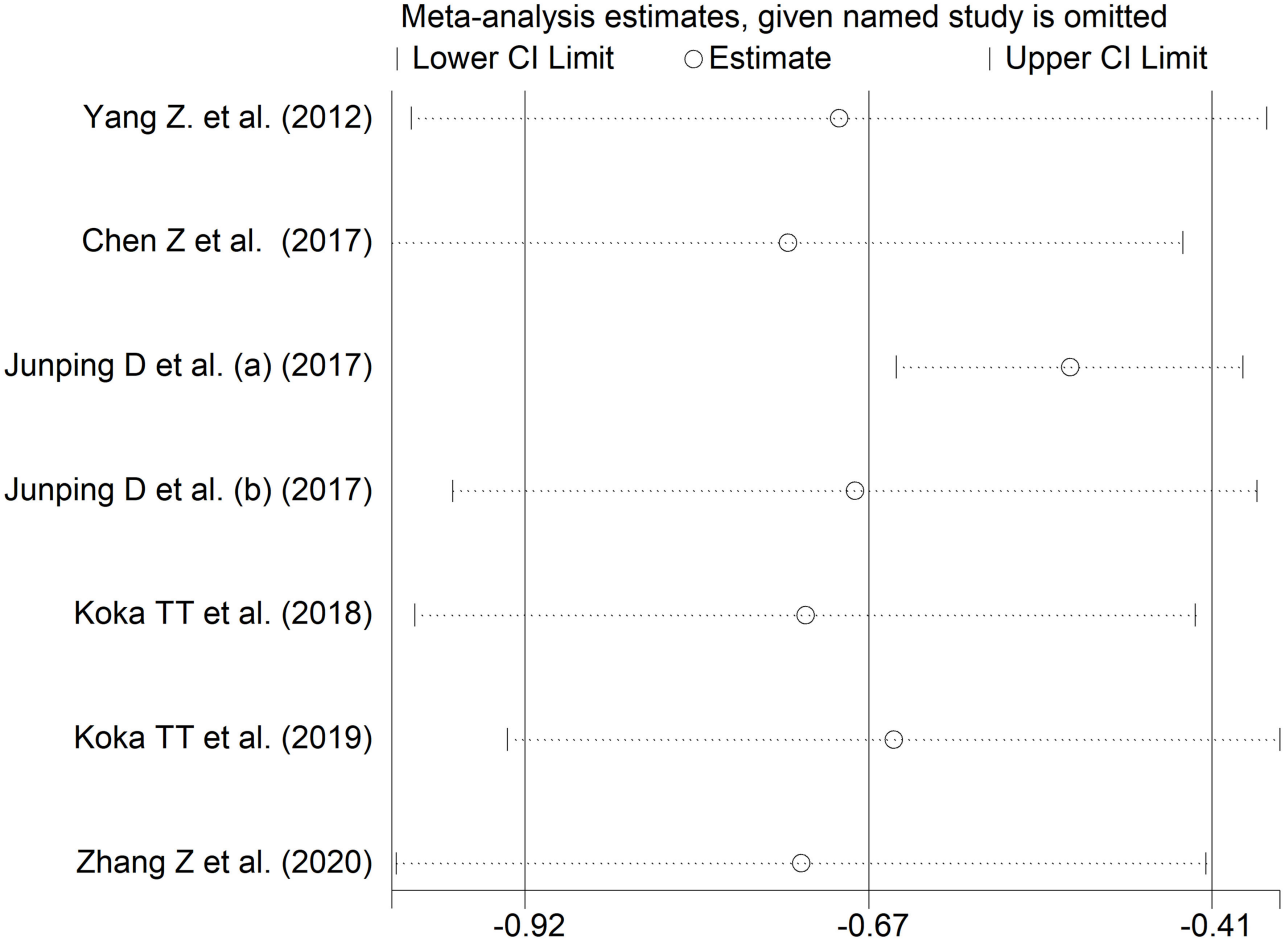
Sensitivity analysis of the association between direct bilirubin and rheumatic diseases.

The forest plot showed that patients with RDs had significantly lower direct bilirubin concentrations when compared to controls (SMD=-0.67, 95% CI -0.92 to -0.41, p<0.001; I^2^ = 81.7%, p<0.001; **Figure 9**). The results were stable in sensitivity analysis, with the corresponding pooled SMD values ranging between -0.72 and -0.52 (**Figure 10**).

The assessment of publication bias, meta-regression, and sub-group analyses could not be conducted because of the limited number of studies. Consequently, the certainty of evidence was downgraded to very low (rating 1).

### Indirect (unconjugated) bilirubin and rheumatic diseases

Seven studies including eight comparator groups investigated indirect bilirubin concentrations in a total of 735 patients with RDs (mean age 46 years, 67% females) and 981 healthy controls (mean age 45 years, 65% females) (34, 38, 39, 42, 43, 45, 48). Five studies were conducted in China (34, 38, 39, 45, 48), and two in Turkey (42, 43). Two study groups included patients with rheumatoid arthritis (39, 43), one with systemic lupus erythematosus (34), one with myositis (38), one with osteoarthritis (39), one with Behcet disease (42), one with primary Sjögren syndrome (45) and one with psoriatic arthritis (48). The study design was retrospective in four studies (39, 42, 45, 48), prospective in one (34), and unknown in the remaining two (38, 43).

**Figure 11:**
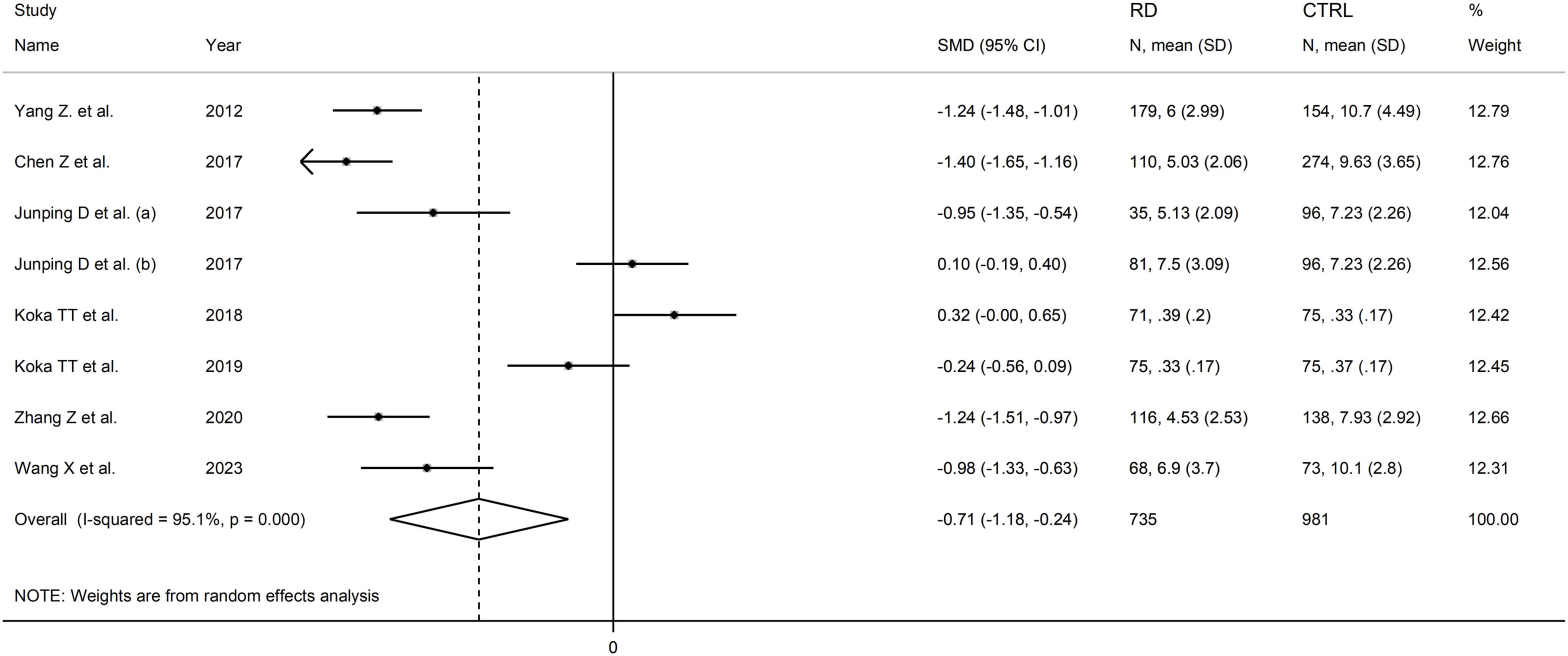
Forest plot of studies investigating indirect bilirubin in healthy controls and patients with rheumatic diseases.

**Figure 12:**
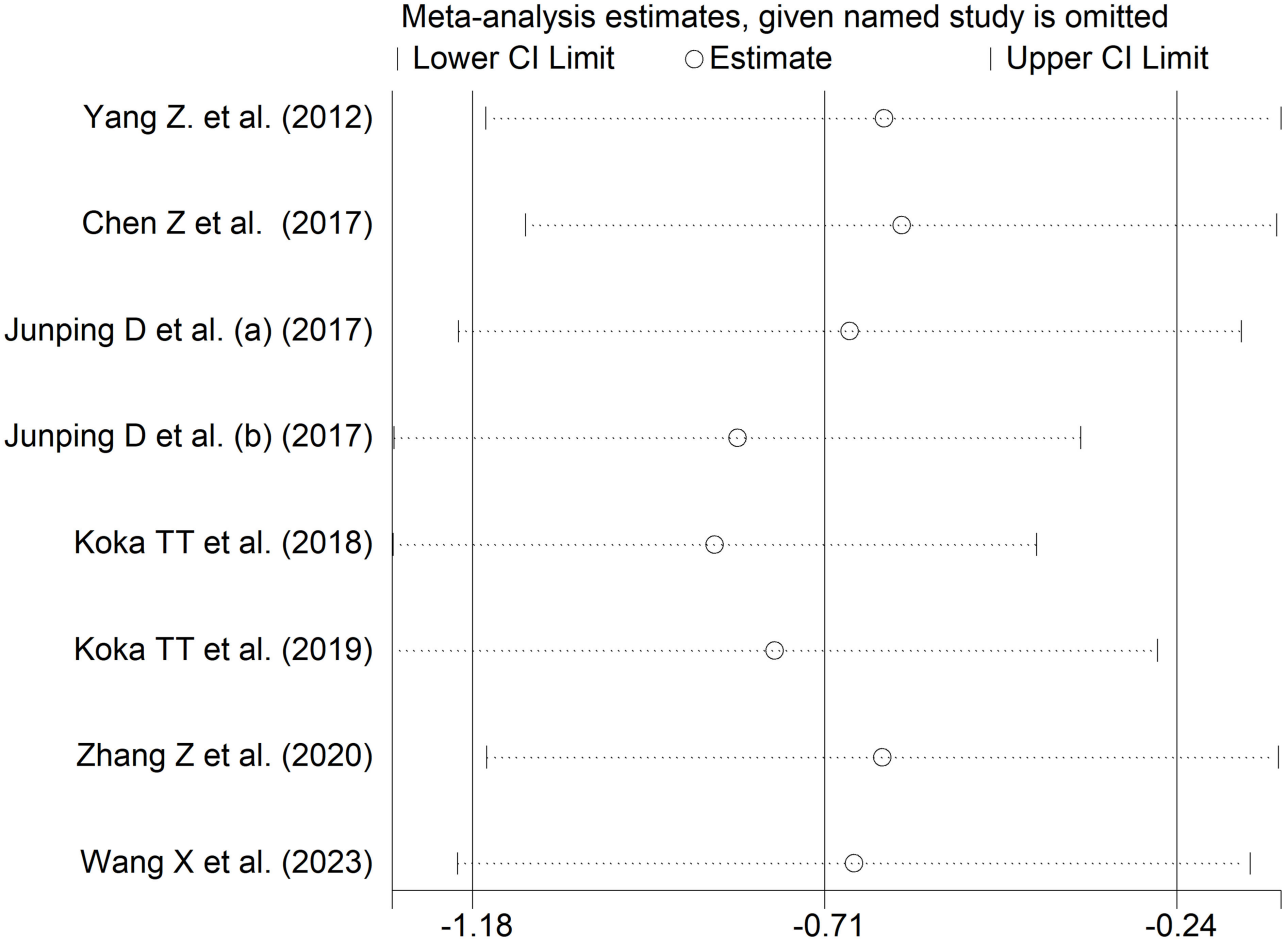
Sensitivity analysis of the association between indirect bilirubin and rheumatic diseases.

The forest plot showed that patients with RDs had significantly lower indirect bilirubin concentrations when compared to healthy controls (SMD=-0.71, 95% CI -1.18 to -0.24, p=0.003; I^2^ = 95.1%, p<0.001; **Figure 11**). Sensitivity analysis confirmed the stability of the results, with the corresponding pooled SMD values ranging between -0.82 and -0.60 (**Figure 12**).

The assessment of publication bias, meta-regression, and sub-group analyses could not be performed because of the limited number of studies. Consequently, the certainty of evidence was downgraded to very low (rating 1).

## Discussion

The reported significant reductions in circulating bilirubin concentrations in patients with RDs when compared to healthy controls, particularly the indirect (unconjugated) fraction which possesses anti-inflammatory and antioxidant effects *in vitro* and *in vivo* and the total fraction, which includes the unconjugated fraction, suggest a significant dysregulation in redox balance and inflammatory pathways in these patients. Notably, the results of our systematic review and meta-analysis also suggest that such alterations can be easily captured through the measurement of this endogenous end product of heme metabolism, which has been part of routine hematological and liver assessment in clinical practice for over 60 years (18, 51). Sensitivity analyses confirmed the stability of the results of the meta-analysis. Meta-regression analyses, only possible for total bilirubin, did not show any significant associations between the effect size and age, male to female ratio, sample size, AST, or ALT, and a non-significant trend with ESR. Subgroup analyses, also specifically conducted on total bilirubin, showed that the SMD was significant across several types of RDs belonging to the classic polygenic autoimmune type (e.g., rheumatoid arthritis, systemic lupus erythematosus, primary Sjögren syndrome, and myositis). In further analyses, the pooled SMD was statistically significant in studies conducted in China but not in other countries but was similarly significant in prospective and retrospective studies.

One of the landmark experiments demonstrating the antioxidant effects of bilirubin was reported by Stocker et al. in 1987. The investigators speculated that bilirubin contains an extended system of conjugated double bonds and a reactive hydrogen atom with potential antioxidant properties. In their *in vitro* experiments, bilirubin at micromolar concentrations was able to efficiently scavenge peroxide radicals generated in homogenous solution or multilamellar liposomes (52). However, prior to these experiments, there was evidence that bilirubin was able to prevent the oxidation of vitamin A and unsaturated fatty acids (53). Subsequent studies reported a protective effect of bilirubin against the development of atherosclerosis and inverse associations with biomarkers of inflammation, including C-reactive protein (54), monocyte chemoattractant protein-1 (55), tumour necrosis factor-α (56), and interleukin-6 (13, 56). There is also evidence that bilirubin can exert immunomodulatory effects, e.g., inhibiting the complement cascade by interrupting the binding of the C1 complex to the antibody (57). These effects were confirmed in *in vivo* experiments where the infusion of unconjugated bilirubin in rats primed for intravascular hemolysis through complement fixation reduced the lysis of blood cells (58). Unconjugated bilirubin has also been shown to interact with macrophages (59), inhibit the cell surface expression of MHC II class molecules on antigen-presenting cells (60), and inhibit lymphocyte proliferation (61). Therefore, the observation of a relative deficiency in unconjugated bilirubin in the presence of RDs might suggest a consumption process driven by scavenging peroxide radicals and/or inflammation although the exact mechanisms responsible require further investigation (15).

While the results of our study suggest the potential utility of bilirubin as a biomarker of inflammation and oxidative stress in RDs, they also raise the intriguing possibility of using bilirubin therapeutically to combat the dysregulated redox and inflammatory pathways in these conditions. Pending further research to corroborate this hypothesis there is evidence that several interventions can increase bilirubin concentrations. For example, acute weight loss in overweight and obese subjects has been associated with a progressive increase in serum bilirubin concentrations, with each 1% weight loss associated with a mean 0.21 μmol/L increase in men (p<0.001) and 0.11 μmol/L increase in women (p<0.001) (62). Notably, the investigation of possible associations between the effect size of between-group differences in bilirubin concentrations and body mass index using meta-regression could not be conducted in our study as only four articles reported information on this anthropometric parameter (36, 39, 41, 47). A higher intake of flavonoid-rich fruit and vegetables and intensive regular exercise have also been shown to increase bilirubin concentrations (63, 64). Finally, several pharmacological agents, e.g., non-steroidal anti-inflammatory drugs, statins, fibrates, and niacin have been shown by increase bilirubin following the activation of heme oxygenase-1 (65). Accurately designed prospective studies are now warranted to determine whether the elevations in bilirubin concentrations induced by these interventions translate into tangible antioxidant and anti-inflammatory effects and, consequently, into a clinical improvement in patients with RDs.

An interesting observation in subgroup analysis for total bilirubin was the presence of significant between-group differences in studies conducted in China but not in those conducted in other countries, suggesting the presence of ethnic differences in bilirubin concentrations. Previous studies conducted in USA have reported significant ethnic-related differences in bilirubin concentrations, with African Americans having lower bilirubin concentrations when compared to white Caucasians and Mexican Americans (66). Clearly, more research is needed to confirm the reported reductions in bilirubin concentrations in RDs patients of different ethnicity and geographical location.

Our study has several strengths, including the assessment of bilirubin concentrations in different types of RDs within the autoinflammatory-autoimmune continuum including autoinflammatory, mixed-pattern, and autoimmune diseases (67, 68), the assessment of associations between the effect size and several study and patient characteristics, and a rigorous evaluation of the risk of bias and the certainty of evidence. Furthermore, sensitivity analysis ruled out the effect of individual studies on the overall effect size. Important limitations include the focus of the studies identified in our search on a restricted number of RDs (systemic lupus erythematosus, polymyositis, dermatomyositis, psoriatic arthritis, systemic sclerosis, rheumatoid arthritis, osteoarthritis, Takayasu arteritis, Behcet disease, primary Sjögren syndrome, and spondyloarthritis), and the paucity of evidence from studies conducted in specific geographical locations, particularly Europe and North and South America. These issues require further study given the established evidence of differences in inflammatory response across different types of RDs and ethnic groups (69–74). Additional issues worth investigating in future studies are the diagnostic accuracy of bilirubin concentrations in RD patients with co-existing liver disease, a phenomenon recently described in patients with autoimmune conditions (75), and the effects of fasting and specific dietary patterns (76–78).

In conclusion, our systematic review and meta-analysis has shown the potential utility of bilirubin as an easily measurable and widely available biomarker of RDs. However, additional research is required to confirm these observations and determine whether this endogenous antioxidant and anti-inflammatory agent can enhance the diagnostic capacity of current biomarkers and other clinical parameters in patients with different types of RDs and ethnicity and co-existing liver disease and, potentially, serve as a therapeutic strategy in this group.

## Data availability statement

The raw data supporting the conclusions of this article will be made available by the authors, without undue reservation.

## Author contributions

AZ: Writing – review & editing, Writing – original draft, Visualization, Validation, Methodology, Investigation, Formal analysis, Data curation, Conceptualization. AM: Writing – review & editing, Writing – original draft, Validation, Supervision, Investigation, Data curation.
